# Ultra-Narrow-Band Filter Based on High Q Factor in Metallic Nanoslit Arrays

**DOI:** 10.3390/s20185205

**Published:** 2020-09-12

**Authors:** Ling Guo, Mengran Guo, Hongyan Yang, Jun Ma, Shouhong Chen

**Affiliations:** 1 School of Electronic Engineering and Automation, Guilin University of Electronic Technology, Guilin 541004, China; guoling@guet.edu.cn (L.G.); G_mengran@163.com (M.G.); majun@guet.edu.cn (J.M.); 2 Guangxi Key Laboratory of Opto-Electronic Information Processing, Guilin 541004, China; 3 Guangxi Key Laboratory of Automatic Detecting Technology and Instruments, Guilin 541004, China

**Keywords:** surface plasmon, nonlinear optics, hybrid SP resonance, high *Q* resonance

## Abstract

Here we propose a novel high *Q* ultra-narrow-band filter in the optical regime. Multiple high *Q* resonances are achieved in ultra-thin metallic nanoslit arrays on stacked low index–high index dielectric (LID–HID) substrate. Based on the cooperative effect of suppressed modes and transmission modes, the high spectral resolution of transmission peaks is obtained. The number and *Q* factor of transmission peaks can be freely manipulated by a simple combination of the stacked LID–HID. It is demonstrated that the linewidths of the transmission peaks can be reduced down to the extreme limit of 1 nm and the *Q* factor is up to 700 by optimizing the structure parameter of the three-layer LID–HID. The results provide a theoretical basis to design a multi-band nanophotonic device with a high *Q* factor and have potential applications in the next generation of high-resolution plasmonic biosensing and filtering.

## 1. Introduction

Nanomaterials and nanostructures have been the research focus in recent years for their potential in various fields, such as lasers [1,2,3], medicine [4,5,6,7], renewable energy [8], and communication [9,10,11,12]. The metallic nanostructure is standing out due to its advantages of small dimension, high integration, subwavelength field confinement, and broad-spectrum. It provides a new way to realize the miniaturization of photonic devices and integrated optics. However, compared to the all-dielectric resonators [13], the realization of high *Q* value in the metallic structure remains a big challenge due to the large metal ohmic loss in optical regime and radiation loss, which is limiting the performance of the plasmonic structure in many fields. For example, high *Q* resonance is helpful in improving the sensitivity for ultra-high sensitive sensing. The figure of merit, which is an important factor to assess the detention limit, is strongly restricted by the spectral width of the resonance and can be improved by a high *Q* resonance with narrow linewidth. On the other hand, high *Q* resonance can be used as an ultra-narrow band filter, which has application value in high-precision optical signal processing, generation of narrow linewidth source and so on.

In recent years, many research groups around the world have been working on decreasing the loss and improving the *Q* factor of the plasmonic structure. Generally, there are two major methods based on mode coupling to solve this problem in this field. One is fano resonance, which is the interference between a broader-band resonance mode and a narrower-band resonance mode, or two neighbored overlapping resonance modes [14,15]. Based on the fano resonance, the asymmetric nanostructure is suggested; by mediating dissipative losses in anti-symmetric cavity resonance modes, sharper resonance with a high *Q* factor (Q<30) can be achieved compared to the symmetric structure [16,17,18]. Another effective method to improve the quality factor is based on a low-loss surface plasmon mode in a multilayer system. In such a system, every single interface of metal can sustain bound surface plasmon polaritons (SPPs). When the separation between adjacent interfaces is comparable to, or smaller than, the decay length of the interface mode, interactions between the interface modes give rise to coupled modes. To obtain a low-loss coupled mode, various new plasmonic structures have been widely studied. It is reported by the Zhijun Sun group that metallic nanoslit arrays can achieve a narrow-band transmission peak with Q~56 based on various hybridized bound plasmon modes [19,20]. In 2018, the Lei Wang group proposed a periodic plasmonic structure with minimized feature sizes [21] to reduce the radiative loss, and resonance with linewidth down to 3 nm (Q~320) is achieved due to the long-lived SPP mode of the grating. In 2019, a plasmonic structure based on surface lattice resonant modes is presented by the Guangyuan Li group [22], and it can achieve a quality factor of 147 under oblique incidence. 

The above methods are based on either the interaction between two resonance modes or the coupling effect of interface modes in multiple layers. In this work, combining the advantages of the above two methods, the cooperative effect of three resonance modes, i.e., two-hybrid surface plasmon (SP) modes with low loss that suppress transmission and a cavity mode that supports transmission, is proposed. By utilizing the suppression modes to tailor the transmission spectrum, transmission peaks with extremely narrow linewidth can be achieved. A plasmonic structure composed of ultra-thin metallic nanoslit arrays and stacked low index-high index dielectric (LID–HID) is put forward. Using the finite-difference time-domain method (FDTD), the simulation demonstrates that the linewidths of the transmission peaks can be reduced even down to ∼ 1 nm and the *Q* factor is up to 700 in the proposed structure with a three-layer LID–HID. The results may be applied in designing multi-band devices with high *Q* factor, high sensitive biosensing, high *Q* filtering, nanolaser, advanced photonic devices, and so on.

## 2. Model and Theoretical Analysis

**Figure 1:**
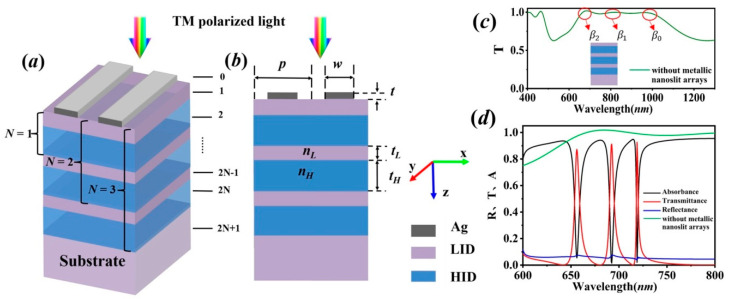
(**a**) 3D view; (**b**) cross-section view of configuration of the ultra-thin metallic nanoslit arrays on N-layer stacked LID–HID above the LID substrate (N is the layers of LID–HID, here take N = 3 as an example); (**c**) the transmission spectrum of 3-layer (N = 3) stacked LID–HID structures without metallic nanoslit arrays; (**d**) the spectra of the ultra-thin metallic nanoslit arrays on 3-layer (N = 3) stacked LID–HID above the LID substrate.

Figure 1 schematically illustrates the configuration of the ultra-thin metallic nanoslit arrays on the *N*-layer of stacked LID–HID above the LID substrate. *N* is the layers of LID–HID, and here takes N=3 as an example, p is the period of the metallic nanoslit array, w is the width of the metal stripe, and t is the metal thickness. The thickness and refractive indices of the LID and HID are denoted as $t_{L}$, $t_{H}$ and $n_{L}$, $n_{H}$, respectively. We are more concerned about the phenomenon that occurs for the transverse magnetically (TM) polarized light, as only TM polarized light can excite the surface plasmon polaritons waves. Thus in the simulation, the TM polarized light is set to be normally incident on the ultra-thin metallic nanoslit arrays. The metal is assumed to be silver and the multiple Lorentz model is adopted to calculate the permittivity here [23]. This is because the multiple Lorentz model is more consistent with the experimental data in the visible and near-infrared range than the Drude model, since it is not only free electrons that contribute to the dielectric constant of metals. Therefore, the permittivity of metal is characterized by the multiple Lorentzian model:

(1) $$\varepsilon (\omega )=\varepsilon_{\infty }+\sum_{k=1}^{6}\frac{\Delta \varepsilon_{k}}{a_{k}{\left(i\omega \right)}^{2}-b_{k}(i\omega )+c_{k}}$$

where $\varepsilon_{\infty }$ is the value of permittivity in the limit of infinite frequency, $\Delta \varepsilon_{k}$ is the strength of each resonance, and $a_{k}=1$, $b_{k}=2\delta_{k}$ ($\delta_{k}$ is the damping factor). In addition, $c_{k}=\omega_{k}^{2}$ ($\omega_{k}$ is the resonant frequency) are fitting coefficients. Obviously, the $b_{k}$ values are affecting the damping factor that causes metal absorption loss, and has a major influence on the imaginary part of the complex dielectric function, which is restricting the quality factors of the resonances. For the silver (Ag) used in this work, six terms of fitting with optimal values [23,24] were applied with $\varepsilon_{\infty }=1$, $\Delta \varepsilon_{k}=$ (1,756.471, 135.344, 258.1946, 22.90436, 1,749.06, 11,756.18), $a_{k}=1$ for all, $b_{k}=$ (0.243097, 19.68071, 2.289161, 0.329194, 4.639097, 12.25), and $c_{k}=$ (0, 17.07876, 515.022, 1,718.357, 2,116.092, 10,559.42). It is proved [23] that the multiple Lorentz model of Ag is consistent with the experimental data in a wide wavelength range.

In this structure, the *N*-layer of the LID–HID stack is considered as a multilayered waveguide. To discuss the multilayer stack, the layers are numbered i=0, 1, 2,⋯2N−1, 2N, 2N+1. The area i=0 is the light-incident region and the area of i=2N+1 is the substrate. $\beta_{i}=k_{0}n_{i}\sin\theta_{i}$ is the component of the propagation constant along x direction where $k_{0}=2\pi /\lambda$, $n_{i}$ is the refractive index and $\theta_{i}$ is an incident angle in the *i*-th layer. Using the theory of thin film optics, the elements of the characteristic matrix of the *i*-th layer are given as [25].

(2) $$\begin{array}{l} m_{11,i}=\cos(k_{0}t_{i}P_{i})\\ m_{12,i}=-j\sin(k_{0}t_{i}P_{i})/P_{i}\\ m_{21,i}=-j\sin(k_{0}t_{i}P_{i})/P_{i}\\ m_{22,i}=\cos(k_{0}t_{i}P_{i}) \end{array}$$

where $j=\sqrt{-1}$, $t_{i}$ is the thickness of the *n*th layer, $P_{i}$ is defined as $P_{i}=\sqrt{n_{i}^{2}-\beta_{i}^{2}/k_{0}^{2}}$. The total characteristic *ABCD* matrix of the *i*-layer structure is the product of the individual layer characteristic matrices and provides a relationship between the electric fields in the superstrate and the substrate. The reflected and the transmitted electric-field intensities at the boundaries of the multilayer stack are:

(3) $$\left[\begin{array}{l} -1\;A+BP_{2N+1}\\ P_{0}\;C+DP_{2N+1} \end{array}\right]\left[\begin{array}{l} E_{r0}\\ E_{t0} \end{array}\right]=\left[\begin{array}{l} E_{i0}\\ P_{0}E_{i0} \end{array}\right]$$

The eigenvalue equation of the multilayer waveguide is then:

(4) $$\left[\begin{array}{l} -1\;A+BP_{2N+1}\\ P_{0}\;C+DP_{2N+1} \end{array}\right]=0$$

which is the dispersion relation for waveguide modes that can be supported by the multilayer stack. There are exactly *N* guided modes with different field distributions in the allowed band [26], e.g., from Figure 1c, there exist 3 guided modes in the 3-layer (N=3) stacked LID–HID structures without the metallic nanoslit arrays, whose propagation constants are labelled as $\beta_{0}$, $\beta_{1}$, $\beta_{2}$. The guided modes in the multilayer stack with propagation constants $\beta_{m}(m=0,1,\cdots ,N-1)$ are coupling with the SP mode at the thin metal strip in each unit, leading to the hybrid SP modes that are suppressing the transmission. Also, the optical tunneling through the slits is resulting in the weakly confined cavity modes, which are supporting the transmission. Therefore, based on the cooperative effect of suppressed modes and transmission modes, i.e., by modulating the hybrid SP modes to tailor the edges of peaks resulted from cavity mode, transmission peaks with high-quality factors can be achieved.

In Figure 1d, the transmission (T), reflection (R) and absorption spectra are plotted with p=400 nm, w=320 nm, t=40 nm, $t_{L}=100\;\mathrm{nm}$, and $t_{H}=200\;\mathrm{nm}$. The simulation is performed with the finite-difference time-domain method (Rsoft Fullwave). In our design, the metal layer is used to restrict non-resonant transmission of light, while allowing plasmon-assisted transmission in resonant conditions. The metal thickness should not be set too thick because it will suppress the resonant transmission by weakening confinement of SPs in the slits [20,27]. On the other hand, the metal should not be set too thin as the incidence light more directly penetrate through the metal layer instead of coupling into the hybrid modes. Therefore, the metal thickness is set to 40 nm. Similarly, the metal width *w* will affect weakly confined cavity modes in the slit, it should not be set too wide since the transmission band would be broadened. In addition, it should not be set too narrow because it will create difficulty in fabricating the ultra-narrow slit; here *w* is set 320 nm. Furthermore, the LID–HID stack is introduced underneath the metallic structure, so that SP modes at the ultra-thin metal layer transform into low-loss hybrid SP modes to support high-quality resonances, and it is verified that $t_{L}=100\;\mathrm{nm}$ and $t_{H}=200\;\mathrm{nm}$ can achieve low-loss hybrid SP modes [19,20]. As we are more concerned with the property of the structure in the optical regime, the period is therefore set at 400 nm. From Figure 1d, it is shown that three ultra-narrow peaks and dips are present in transmission spectra corresponding to the weakly confined cavity modes and the hybrid SP modes, respectively. Obviously, without the metallic nanoslit arrays, the hybrid SP modes and weakly confined cavity modes can’t be excited in the stacked structure. The reflection spectrum has roughly complementary profiles with the transmission spectrum. It is suggested and proved by the absorption spectrum that absorbance for the resonances is relatively low.

## 3. Simulation and Discussion

**Figure 2:**
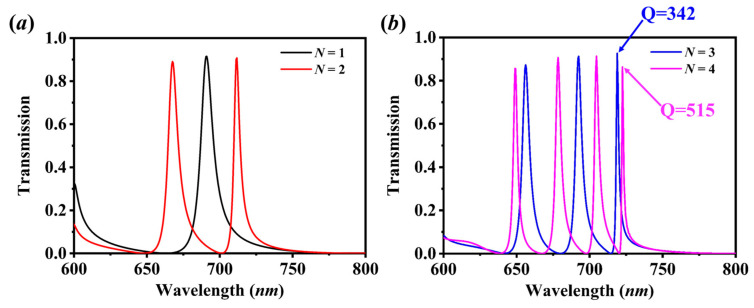
Transmission spectra of the ultra-thin metallic nanoslit arrays based on an N-layer stacked LID-HID with (**a**) N=1 and N=2 (**b**) N=3 and N=4.

Figure 2 shows the classical transmission spectra of the ultra-thin metallic nanoslit arrays on a 3-layer LID–HID above the LID substrate, in which the period p=400 nm, the width of the metal stripe w=320 nm, the metal thickness t=40 nm, the LID thickness $t_{L}=100\;\mathrm{nm}$, and the HID thickness $t_{H}=200\;\mathrm{nm}$. For N=1, shown as the black line in Figure 2a, i.e., for only one layer of LID–HID, the transmission peak with the full width at half maximum (FWHM) of 8 nm at 691 nm is due to the cooperative effect of a transmission mode and two suppressed modes located on both sides of the peak [19,20]. For the structure with N=2, it is observed that there are two transmission peaks positioned at 668 nm and 712 nm in the optical regime. Similarly, when increasing the layers of LID–HID (noted as *N*) further, as shown in Figure 2b for N=3 and N=4, three (at 656 nm, 692 nm, 719 nm) and four (at 649 nm, 678 nm, 705 nm, 723 nm) transmission peaks are appearing, respectively, in the spectra. It is noted that by increasing the layers *N*, new SP modes, including transmission modes and suppression modes, can be excited in the structure and produce the new ultra-narrow peaks. Also, widths of multiple peaks are reducing effectively with increasing *N*. For N=3, it is observed that the minimum FWHM of the peaks at 719 nm (with a large transmittance ~0.9) is down to ~2.1 nm, and the quality factor (~342) is improved 3–4 times compared to the *Q* factor for N=1. When N=4, the minimum FWHM of the peak at 722 nm is down to ~1.4 nm and the quality factor (~515) is improved five times compared to the case of N=1. This is because the positions of the suppression modes located on both sides of the peaks are getting closer as the number of suppression modes is increasing, therefore, the linewidth of peaks can be reduced effectively to improve the *Q* factor. It is concluded that it is not only possible to design the easily fabricated plasmonic structure with multiple narrow-band transmission peaks, but it also provides a new route in improving the *Q* factor.

**Figure 3:**
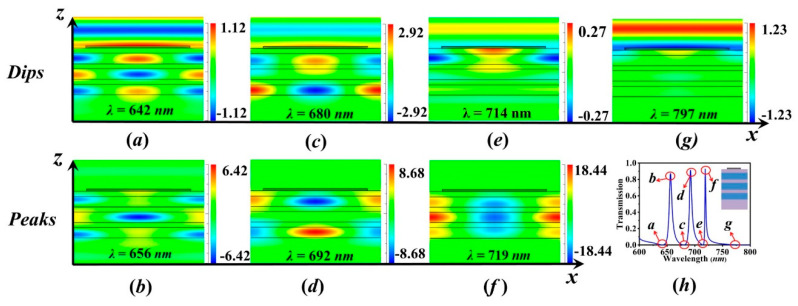
Magnetic distributions (*H*_y_) for structure with N=3, p=400 nm, w=320 nm, t=40 nm, $t_{L}=100\;\mathrm{nm}$, $t_{H}=200\;\mathrm{nm}$, $n_{L}=1.5$, $n_{H}=2$ at the resonant dips (**a**) λ=642 nm, (**c**) λ=680 nm, (**e**) λ=714 nm, (**g**) λ=797 nm and peaks (**b**) λ=692 nm, (**d**) λ=692 nm, (**f**) λ=719 nm in the (**h**) transmission spectrum.

To identify the resonant modes, magnetic distributions ($H_{y}$) at the resonant dips and peaks labeled in Figure 3h for the structure N=3 are calculated in Figure 3. For the suppressed mode λ=797 nm in Figure 3g, whose magnetic field is distributed in the metal strip, it is the anti-symmetric bound SP mode at the thin metal film gratings, which is reported in [27]. For the other suppressed modes with nearly zero transmission, illustrated in Figure 3a,c,e, it is observed in the z-direction that the fields are confined within the entire structure composed of the metal stripe and stacked LID–HID, and shows the characteristics of hybrid SP waveguide modes. From distributions of the fields, the resonant modes at λ=714 nm, λ=680 nm and λ=642 nm, are identified as zeroth order ($TM_{0}$), first order ($TM_{1}$) and second-order ($TM_{2}$) hybrid SP waveguide modes according to the zero-field nodes in the z-direction. For the transmission modes shown in Figure 3b,d,f, the fields are mainly distributed in stacked LID–HID dielectric cavity under the metal stripe. The resonant modes are enhanced strongly and showed a significant transmission. From the zero-field nodes in the *z*-direction, the resonances at λ=656 nm, λ=692 nm and λ=719 nm are noted as *zero*th-order, first-order, and second-order of dielectric cavity modes. While in the *x*-direction, the fields of the hybrid SP waveguide modes and the cavity modes demonstrate two nodes with zero-field within a period, and it is inferred that these modes are similar to the 2-order Bloch-wave effect in the layered media with an infinite period. As the hybrid SP waveguide modes and the slit cavity modes are located close in the frequency, the suppressed modes can tailor the edges of transmission peaks, and ultra- narrowband transmission peaks are achieved.

**Figure 4:**
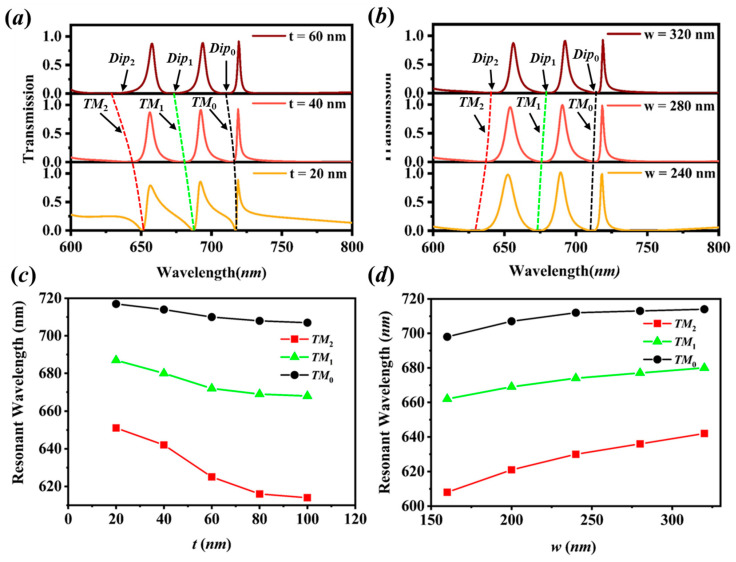
(**a**,**b**) Transmission spectra of the proposed structure (N=3, p=400 nm, $t_{L}=100\;\mathrm{nm}$, $t_{H}=200\;\mathrm{nm}$, $n_{L}=1.5$, $n_{H}=2$) for various t with w=320 nm and various w with; (**c**,**d**) dependences of resonant wavelengths of the suppressed hybrid SP waveguiding modes noted as $TM_{0}$, $TM_{1}$ and $TM_{2}$ on t and w.

For further investigation of the mechanisms on the multiple transmission peaks, transmission spectra of the proposed structure of N=3 for various *t* are calculated in Figure 4a. It is observed that the hybrid SP waveguide modes noted as $TM_{0}$, $TM_{1}$, and $TM_{2}$ have a blue shift, while the transmission modes in the multi-high/low-index dielectric cavity move little with increasing metal thickness *t*. It is inferred that the metal thickness *t* has a major impact on the hybrid SP waveguide resonances, while it has a weak influence on the transmission modes in the dielectric cavity. In fact, the effect of metal thickness *t* is similar to the interaction of thin metal grating on the substrate. With increasing the metal thickness *t*, the real part of the effective index of the hybrid SP modes would decrease [27], leading to the blue shift of resonant dips. From Figure 4c, it is clear that the dip position of $TM_{2}$ mode decreases sharply, the position of $TM_{1}$ mode reduces moderately, and the $TM_{0}$ mode moves slowest. This is because the field of $TM_{2}$ mode in the metal is relatively stronger while that of $TM_{0}$ mode is relatively weak, which can be seen from the Figure 3a,c,e, therefore, the influence of metal thickness on $TM_{2}$ mode is the largest. Meanwhile, it is obvious that the FWHM of the peaks at t=20 nm is widened much more compared those of others; this is because the metal is thin enough for the light to be transmitted directly through the metal. Figure 4b,d illustrate the effect of the metal width w. From Figure 4b, we can see that the hybrid SP resonances have a red shift with increasing the metal width w. This is because the wider metal strip means a narrower slit, the effect of slit width between the metal strips is similar to that of the metal-insulator-metal (MIM) waveguide structure. By increasing the metal width w, i.e., decreasing the slit width, the real part of the effective index of the hybrid SP modes would increase [28], leading to the red shift of resonant dips. In Figure 4d, the dip positions of $TM_{0}$, $TM_{1}$, and $TM_{2}$ are redshifted near-linearly. According to the slope, it is demonstrated that the high order of the hybrid SP resonance moves more quickly than the lower order resonance, which means that the dip on the left of the peak is redshifted more rapidly than the dip on the right. Therefore, the FWHM of the peaks are narrowing with increasing the metal width w.

**Figure 5:**
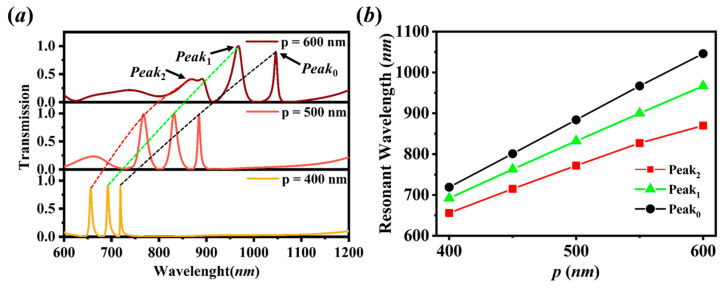
(**a**) Transmission spectra of the proposed structure (N=3, w=320 nm, t=40 nm, $t_{L}=100\;\mathrm{nm}$, $t_{H}=200\;\mathrm{nm}$, $n_{L}=1.5$, $n_{H}=2$) for various period p. (**b**) dependences of resonant wavelengths of the transmission modes noted as $Peak_{0}$, $Peak_{1}$ and $Peak_{2}$ on period p.

To investigate the effect of the periodicity, the transmission spectra of the proposed structure N=3 for various periods are calculated in Figure 5a. It is demonstrated that the peak positions are redshifted with an increase of the period *p*. This is because the resonant modes show the second-order Bloch-wave nature and the resonant wavelength can be estimate as λ≈Re(neff)⋅p [19,26]. With increasing the period *p*, the resonant wavelength becomes larger and redshifted. It is proved in Figure 5b that the resonant wavelengths of the peaks are linear with the period and the low-order of the resonant mode moves more quickly than the high-order does from the analysis of slope. Meanwhile, the widths of the peaks are broadened and the interval between the peaks is larger compared to the small period. This is because it is noted that, for a large period (p=600 nm), the transmittance of $Peak_{2}$ is reduced sharply, and the *Peak*_2_ is becoming wider and deforms, and even disappears if we further increasing the period. This is because *Peak*_2_ is near the cut-off frequency and would disappear above the cut-off [19], thus we need to redesign the dimensions of structure for a large period.

**Figure 6:**
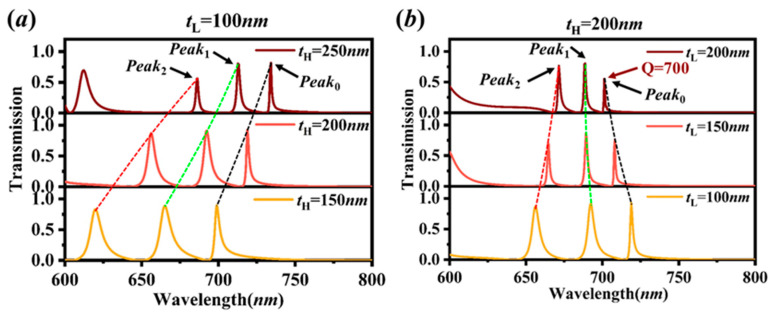
(**a**,**b**) Transmission spectra of the proposed structure (N=3, w=320 nm, t=40 nm, p=400 nm, $n_{L}=1.5$, $n_{H}=2$) for various HID thickness $t_{H}$ and LID thickness $t_{L}$.

The investigation on HID thickness $t_{H}$ and LID thickness $t_{L}$ of the proposed structure is calculated in Figure 6. From Figure 6a, with increasing the HID thickness $t_{H}$, it is observed that the peaks’ positions have a red shift due to the increase of the real part of the effective index of the resonant modes [19]. Furthermore, as the dips further compress the transmission band, the peak widths decrease distinctly, meanwhile, the distance between the peaks is reduced. It is also found that, for large $t_{H}$, the new peaks due to the resonances of higher-order waveguide modes move into the spectral range [20], and would appear at a shorter wavelength, e.g., at 612 nm for $t_{H}=250\;\mathrm{nm}$.

Similarly, with increasing the LID thickness $t_{L}$, the real *N_eff_* decreases for the *TM*_0_ and *Peak*_0_ modes, but increases for the *TM*_2_ and *Peak*_2_ modes and varies little for *TM*_1_ and *Peak*_1_ modes [19]. Therefore, we can see from Figure 6b that the *Peak*_2_ has an obvious red shift, *Peak*_1_ only redshifts slightly while *Peak*_0_ has a blue shift with increasing the LID thickness $t_{L}$. This would make the peaks seem to be concentrated at one point, thus the interval between the peaks is reducing. The peaks’ widths are also reducing effectively, for $t_{H}=200\;\mathrm{nm}$, $t_{L}=200\;\mathrm{nm}$ of Figure 6b, the width of *Peak*_0_ is down to ~1 nm (Q~700), however, the transmittance is descending obviously. Therefore, by modulating the thicknesses of the HID and LID, the *Q* factor can be improved further at the cost of transmittance.

## 4. Conclusions

In summary, ultra-thin metallic nanoslit arrays based on stacked LID–HID above the substrate are proposed and multiple narrow-band transmission peaks are shown in the spectrum due to the cooperative effect of transmission modes and suppressed modes. The transmission modes are formed by the stacked HID-LID layers cavity under the metallic nanoslit array, which supports the high transmission of light. The suppressed modes include two types of resonant modes. One type is the anti-symmetric bound SP mode at the thin metal film; another type is due to the hybrid SP waveguide modes in the metal and LID–HID region that locates on both sides of the transmission mode. Based on the cooperative effect of transmission modes and suppressed modes, the high spectral resolution of transmission peaks can be achieved. It is demonstrated that the linewidths of the transmission peaks can be reduced down to the extreme limit of 1 nm and the *Q* factor is up to 700 by optimizing the structure parameter of three-layer LID–HID. The results provide a theoretical basis to design a multi-band device with a high *Q* factor and have potential applications in the next generation of high-resolution plasmonic biosensing and filtering.

## Data Availability

The data used to support the findings of this study are available from the corresponding author upon request.
